# Improved gesturing in left-hemispheric stroke by right inferior parietal theta burst stimulation

**DOI:** 10.3389/fnins.2022.998729

**Published:** 2022-12-16

**Authors:** Manuela Pastore-Wapp, Dávid M. Gyurkó, Tim Vanbellingen, Dirk Lehnick, Dario Cazzoli, Tobias Pflugshaupt, Stefanie Pflugi, Thomas Nyffeler, Sebastian Walther, Stephan Bohlhalter

**Affiliations:** ^1^Neurocenter, Luzerner Kantonsspital, Lucerne, Switzerland; ^2^ARTORG Center for Biomedical Engineering Research, Gerontechnology and Rehabilitation Group, University of Bern, Bern, Switzerland; ^3^Biostatistics and Methodology, Clinical Trials Unit Central Switzerland, Lucerne, Switzerland; ^4^Department of Health Sciences and Medicine, University of Lucerne, Lucerne, Switzerland; ^5^Department of Psychology, University of Bern, Bern, Switzerland; ^6^Department of Neurology, University Hospital of Bern, Bern, Switzerland; ^7^Translational Research Center, University Hospital of Psychiatry and Psychotherapy of Bern, Bern, Switzerland; ^8^Department of Neurology, University of Zurich, Zurich, Switzerland

**Keywords:** apraxia, gesturing, left hemispheric stroke, non-invasive brain stimulation, transcranial continuous theta burst stimulation, transcallosal connectivity

## Abstract

**Objectives:**

Apraxia is a common syndrome of left hemispheric stroke. A parieto-premotor-prefrontal network has been associated with apraxia, in which the left inferior parietal lobe (IPL-L) plays a major role. We hypothesized that transcranial continuous theta burst stimulation (cTBS) over the right inferior parietal lobe (IPL-R) improves gesturing by reducing its inhibition on the contralateral IPL in left hemispheric stroke patients. It was assumed that this effect is independent of lesion volume and that transcallosal connectivity is predictive for gestural effect after stimulation.

**Materials and methods:**

Nineteen stroke patients were recruited. Lesion volume and fractional anisotropy of the corpus callosum were acquired with structural magnetic resonance imaging (MRI) and diffusion tensor imaging (DTI). Each patient had pseudorandomised sessions with sham or with stimulation over the IPL-R or over the right inferior frontal gyrus IFG-R. Gesturing was assessed in a double-blinded manner before and after each session. We tested the effects of stimulation on gesture performance using a linear mixed-effects model.

**Results:**

Pairwise treatment contrasts showed, that, compared to sham, the behavioral effect was higher after stimulation over IPL-R (12.08, 95% CI 6.04 – 18.13, *p* < 0.001). This treatment effect was approximately twice as high as the contrasts for IFG-R vs. sham (6.25, 95% CI –0.20 – 12.70, *p* = 0.058) and IPL-R vs. IFG-R vs. sham (5.83, 95% CI –0.49 – 12.15, *p* = 0.071). Furthermore, higher fractional anisotropy in the splenium (connecting the left and right IPL) were associated with higher behavioral effect. Relative lesion volume did not affect the changes after sham or stimulation over IPL-R or IFG-R.

**Conclusion:**

One single session of cTBS over the IPL-R improved gesturing after left hemispheric stroke. Denser microstructure in the corpus callosum correlated with favorable gestural response. We therefore propose the indirect transcallosal modulation of the IPL-L as a promising model of restoring interhemispheric balance, which may be useful in rehabilitation of apraxia.

## 1 Introduction

Apraxia is a common syndrome in left unilateral stroke patients, denoting the decreased ability to perform motor skills despite intact primary motor function and coordination. Impaired praxis skills are independent from other cognitive deficits (Osiurak and Bartolo, 2020). Praxis function is essential for gesturing and tool handling in everyday life (Hanna-Pladdy et al., 2003). Clinical assessment of apraxia usually relies on gesturing in two domains: replication on command (pantomime) and on seen gesture (imitation).

Functional imaging studies have revealed a predominantly left hemispheric, parieto-premotor- and prefrontal activation during planning and execution of gesturing for both hands (Hermsdörfer et al., 2001; Chaminade et al., 2005; Mühlau et al., 2005; Frey, 2007; Bohlhalter et al., 2009; Achilles et al., 2019). Furthermore, lesion mapping studies with larger series of stroke patients (Buxbaum et al., 2014; Hoeren et al., 2014; Sperber et al., 2019) identified the left inferior parietal lobe (IPL-L) as a major neural hub for gesturing regardless of the domain involved. This finding is in line with the role of the IPL in the representation of temporal-spatial action (Heilman, 2010). Pantomime further relies on intact mid-temporal areas containing abstract action representation (Lingnau and Downing, 2015; Wurm et al., 2016) and semantic knowledge (Hoeren et al., 2014). For imitation it was proposed that, in addition to IPL-L, a right parietal contribution is needed, when demands on visuospatial exploration and analysis of gestures are high (Park, 2017).

Non-invasive brain stimulation (NIBS) techniques, such as transcranial direct current stimulation (tDCS), repetitive transcranial magnetic stimulation (rTMS), more specifically continuous theta burst stimulation (cTBS), enable investigating praxis skills in healthy subjects and stroke patients with apraxia (Vanbellingen et al., 2014). Non-invasive inhibition with cTBS over the IPL-L (Vanbellingen et al., 2014) and the left inferior frontal gyrus (IFG-L) (Bohlhalter et al., 2011) showed a reversible deterioration of gesturing in healthy subjects. cTBS over the IPL-R improved gestural imitation in healthy subjects (Vanbellingen et al., 2020). The effect correlated with measures of transcallosal connectivity in the splenium corporis callosi to the IPL-L pointing to an interhemispheric facilitation of this area. Furthermore, it was shown in stroke patients, that the excitatory stimulation by tDCS of the lesioned posterior parietal cortex improved the performance of gestural imitation (Bolognini et al., 2015; Ant et al., 2019).

The importance of transcallosal connectivity was first proposed for neglect based on an interhemispheric rivalry model (Kinsbourne, 1977). Accordingly, down-regulating hyperexcitability of the right contra-lesional hemisphere emerged as therapeutic principle in aphasia (Kindler et al., 2012; Thiel et al., 2013). In addition, inhibition of the left was beneficial for neglect (Nyffeler et al., 2008, 2009, 2019; Cazzoli et al., 2012). These findings suggest that restoring the interhemispheric balance by cTBS may also help improving gestural performance in left hemispheric stroke patients. Therefore, the rationale for inhibitory stimulation of the IPL-R using cTBS was to concomitantly facilitate the left IPL, a mechanism that translated into gestural improvement in healthy subjects (Vanbellingen et al., 2020). Inhibition of IFG-R did not produce any effects in this earlier study. Gestural behavior, however, as shown by functional imaging studies (Bohlhalter et al., 2009), was consistently associated with bilateral (though clearly left lateralized) activation not only in IPL but also in the IFG. Accordingly, activity of IFG-R may contribute to interhemispheric inhibition of IFG-L. This area was therefore chosen as an active control condition to assess the specificity of the cTBS effect over IPL-R.

In the present randomized double-blinded sham-controlled proof of concept study we hypothesized that 1) cTBS over the IPL-R will improve gesture performance by diminishing its maladaptive inhibition on the IPL-L and that 2) transcallosal connectivity as measured by diffusion tensor imaging (DTI) in the splenium corporis callosi will predict gestural outcome after stimulation.

## 2 Materials and methods

### 2.1 Patients

Nineteen patients with first stroke involving the left hemisphere were recruited from the Neurocenter Luzerner Kantonsspital (for patient characteristics see Table 1). Inclusion criteria were defined as first time stroke, left hemispheric lesion, apraxia or aphasia, no severe psychiatric condition, no contraindication for magnetic resonance imaging (MRI) or transcranial magnetic stimulation (e.g. metal implants or epilepsy). All patients provided written informed consent prior to the experiment. The study was performed according to the with the Code of Ethics of the World Medical Association (Declaration of Helsinki) and was approved by the local ethics committee. One patient had a CT instead of a magnetic resonance imaging (MRI), therefore lesion overlap and fractional anisotropy (FA) analysis were done with 18 patients.

**TABLE 1:** Patient characteristics.

	Mean ± SD/n (%)	Range
Age (years)	69.3 ± 14.6	34–91
Sex	6 females (31.6%) 13 males (68.4%)	
Time since stroke (days)	29.7 ± 12.0	21–57
**Baseline scores**		
NIHSS	11.1 ± 7.1	2-22
Judgment of line orientation	8.2 ± 3.3	0–13
Boston naming test	6.7 ± 5.0	0–15
Novel tools test	19.8 ± 5.59	7–24
LIMOS	91.3 ± 33.3	51–153
LIMOS upper limb	12.9 ± 5.1	7–23
Relative lesion volume (%) FA values	5.33 ± 7.27 0.46 ± 0.04	0.13–26.89 0.38–0.51

NIHSS, National Institutes of Health Stroke Scale; FA, fractional anisotropy; LIMOS, Lucerne ICF-based multidisciplinary observation scale.

### 2.2 Experimental protocol

**FIGURE 1:**
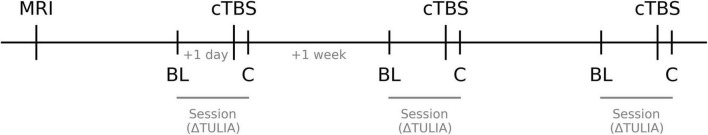
Schematic representation of the experimental protocol. MRI, acquisition of fractional anisotropy with magnetic resonance imaging. BL, Session-related baseline behavioral assessment. C, Session-related control behavioral assessment. cTBS, sham or continuous theta burst stimulation over IPL-R or IFG-R; the order of the stimulation sessions was pseudorandomized with the sequence xyz, xzy, yxz, yzx, zxy or zyx, where x, sham, y, cTBS over right inferior parietal lobe, z, cTBS over right inferior frontal gyrus.

Eighteen stroke patients underwent structural MRI acquisition (36.1 ± 19.5 days) before they entered the experimental protocol. Afterward, each patient had three stimulation sessions of cTBS embedded in two gesture performance assessments in a double-blinded (stroke patients and raters regarding cTBS condition), repeated measures design (Figure 1). Each session started with a gesture performance assessment to control for general rehabilitational effects. After one day, a cTBS was performed either with sham or with stimulation over IPL-R or IFG-R. The gesture performance assessment was repeated right after cTBS to obtain a ΔTULIA value (calculated as control – baseline).

### 2.3 Behavioral outcome

#### 2.3.1 Comprehensive gestural assessments

The gesture performance was assessed with the validated test of upper limb apraxia (TULIA) (Vanbellingen et al., 2010) at the beginning and at the end of each experimental session (BL and C in Figure 1). It consists of 48 items covering the domains imitation and pantomime in three semantic categories (meaningless, communicative and tool related). The performance of each item regarding temporal, spatial and content related errors is rated on a scale ranging from zero to five points. Thus, the TULIA score ranges from zero to 240, (mild apraxia is < 194, moderate apraxia < 130, severe apraxia < 65). The TULIA proved to be a suitable instrument to quantify effects of cTBS on gestural performance in healthy subjects (Bohlhalter et al., 2011; Vanbellingen et al., 2020) and patients with schizophrenia (Walther et al., 2019). Furthermore, as the TULIA has no ceiling effect, it also allows to investigate gestural function in non-apraxic subjects. TULIA scores were rated by two blinded raters (SP, MPW) with high inter-rater reliability (ICC = 0.82). The video-based scoring method allowed a sensitive detection of gestural changes.

#### 2.3.2 ADL functional outcome

Activities of daily life (ADL) was assessed with the Lucerne ICF-based (International Classification of Functioning, Disability and Health-based) Multidisciplinary Observation Scale (LIMOS) on admission and discharge. The 45 LIMOS items measure the level of assistance needed during ADL, with higher scores representing less dependence (Ottiger et al., 2015; Vanbellingen et al., 2016; Van de Winckel et al., 2019). The total score ranges from 45 to 225. The LIMOS has been shown to be more sensitive than the Functional Independence Measure and the Barthel Index (Vanbellingen et al., 2016, 2017).

#### 2.3.3 Control behavioral assessments

Patients completed the ‘orientation test’, i.e., a short version of the Judgment of Line Orientation test (JLO, Mount et al., 2012) a short version of the Boston naming test to control for confounding effects on visual spatial skills and word retrieval (BNT-15, Mack et al., 1992), and the novel tool test (Buchmann and Randerath, 2017) to control for mechanical knowledge at the beginning and at the end of each experimental session (BL and C in Figure 1).

The JLO is a purely visual 30-item test requiring participants were asked to visually examine 11 lines that appear in a standard fan-shaped array at the bottom of the examination sheet. Patients were asked to match angles of two lines, presented on the top of the page. The short version of the JLO consisted of 15-items chosen from the test. To score one point, the angles of both two lines must be matched correctly, thus the short version results in a maximum score of 15, with a cut-off value of 10.

In the Boston naming test the patients were asked to name 15 line drawings. Each correctly named drawing is scored with a point, so that total scores range from 0 to 15, cut off-value is 12.

Mechanical knowledge was assessed with a short form of the Novel Tools Test. The test consisted of 1 practice and 6 test trials using a set of 6 different cylinders and corresponding tools. In each trial a wooden cylinder in a socket were presented. From three tools, the patient had to choose the tool that fitted best to lift the cylinder out of the socket. In each trial two scores were given for the correct tool selection and two scores for the proper use of the tool resulting in a maximum score of 24 (higher values mean better performance, cut-off scores are 17 for females and 18 for males). One point was given for each trial if correct selection and application was reached by trial and error.

#### 2.3.4 Continuous theta-burst stimulation

cTBS was applied by means of a MagPro R30 stimulator (MagPro, Medtronic Functional Diagnostics, Skovlunde, Denmark) connected to a round coil with 60 mm outer radius (Magnetic coil Tranducer, MC-125, Medtronic). A cTBS protocol (Nyffeler et al., 2009) was used, consisting of a continuous train of 801 pulses delivered in 267 bursts. Each burst contains 3 pulses at 30 Hz, with an interburst interval of 100 ms, leading to a total duration of 44 s for one single cTBS train. Target site location was determined according to the international 10-20 EEG system. For IPL-R stimulation cTBS was applied halfway between P4-P8, for IFG-R stimulation the coil was placed over F8 (Bianchi et al., 2015). For each session correct positioning was confirmed by a second examiner (TV, SP and MPW). The coil was placed tangentially over the target area with the handle pointing backards and with the current flowing in a clockwise direction (within the coil) as viewed from above. cTBS was delivered at 100% of the participants’ individual resting motor threshold over IPL-R to also reach deeper structures of the interparietal sulcus and 80% over IFG-R with the same round coil. The lower threshold over IFG-R was chosen to avoid discomfort during stimulation like involuntary facial twitching or tonic muscle contractions (Bohlhalter et al., 2011; Meteyard and Holmes, 2018). Individual resting motor threshold was defined as the lowest stimulation intensity applied over the right primary motor cortex eliciting a visible contraction of the contralateral hand muscle in at least 5 out of 10 consecutive stimuli. Sham stimulation was applied by the same cTBS protocol, however using a sham coil over the vertex (Magnetic Coil Transducer MC-P-B70, Medtronic).

#### 2.3.5 Magnet resonance imaging acquisition

High-resolution T1-structural and DTI were obtained using a 3T Siemens Magnetom Skyra whole-body scanner. We used a diffusion-weighted spin echo, echo-planar imaging sequence to obtain diffusion-weighted scans with 128 directions. The b-values were 0 s/mm2 and 1,000 s/mm2, respectively. Further imaging parameters were: voxel size 1.1 × 1.1 × 4.0 mm3, 34 slices, FoV = 220 mm, TR/TE = 6,800/78 ms, TA 2:45 min. Additionally, anatomical scans were acquired using a T1-weighted three-dimensional (3D) magnetization-prepared rapid acquisition with gradient echo (MPRAGE) sequence (voxel size 1 × 1 × 1 mm3, FoV 230 mm; TR/TE = 1,900/2.43 ms, TA 4:18 min).

#### 2.3.6 MRI processing

##### 2.3.6.1 Lesion mapping, lesion volume

Lesions were manually traced onto the patients’ individual structural MRI images on every transverse slice using the MRIcron^1^, yielding binary lesion maps. Lesion volume was extracted within MRIcron. Images were then normalized into Montreal Neurological Institute (MNI) stereotaxic space using the segmentation algorithm in SPM12^2^. Afterward each lesion was manually checked and, if necessary, corrected. Normalized lesions were smoothed with a 4mm FWHM Gaussian kernel. Lesion overlap maps were generated with the Matlab scripts niiStat^3^ and MRIcroGL^4^. Total intracranial brain volume (TIV) was calculated with the computational anatomy toolbox (CAT12; Gaser et al., 2022) for Statistical Parametric Mapping software (SPM12) (Penny et al., 2006). The lesion volume was divided by TIV providing a relative stroke lesion volume (%) independent from brain size that could be used for further analyses.

##### 2.3.6.2 Diffusion tensor imaging

DTI images were preprocessed using DTIPrep (Liu et al., 2010), a program for automatic image quality control and preparation. Preprocessing included image information check, data cropping, slice-wise, interlace-wise, and gradient-wise intensity artifact correction, eddy current and head motion correction, as well as computing of DTI. For the extraction of the average value of the FA indices in the participant’s native space, the reconstruction method “DTI” proposed by Basser et al. (1994) was performed using DSI Studio^5^ (Yeh et al., 2013). According to the Johns Hopkins University (JHU) white-matter-atlas (Mori et al., 2005) a ROI was placed in the splenium corporis callosi. Average FA-values were extracted for each participant from this ROI. In addition, each tensor was visually inspected to ensure good quality prior to FA map creation. The FA values in the genu and cauda corporis callosi were also tested and showed no correlation with changes in TULIA.

#### 2.3.7 Experimental design and statistical analysis

Descriptive data is presented as mean ± standard deviation (SD) for continuous variables and as frequency (%) for categorical variables.

Linear mixed effects models have been applied to explore the effects of cTBS on gestural performance assessed with the TULIA. BL FA refers to a one-time baseline fractional anisotropy in the splenium corporis callosi, whereas BL TULIA refers to the first TULIA assessment before the first experimental session. To control for learning and rehabilitation effects, period-specific delta values were calculated for each stimulation (e.g. “Post-stimulation TULIA Period 1” – “TULIA Period 1” = “ΔTULIA Period 1”). The primary statistical model included fixed effects for treatment, BL TULIA, FA values, period and sequence and a random effect for subject (allowing for intra-individual comparisons) to explain ΔTULIA as a dependent variable. Sensitivity analyses including further covariates were performed in order to assess the robustness of the model and the estimates. As part of the exploratory analyses, pairwise correlations of patient characteristics at baseline were also determined and considered.

## 3 Results

### 3.1 Clinical baseline characteristics

Of the 19 included patients, 26.3% were severely apraxic (BL TULIA score < 65), 15.9% were moderately (BL TULIA score < 130) and 42.4% were mildly apraxic at baseline (BL TULIA score < 194). Three patients (15.8%) were non-apraxic (BL TULIA score > 194). All patients finished the study without any adverse reaction after cTBS. For details of patient characteristics see Table 1.

**FIGURE 2:**
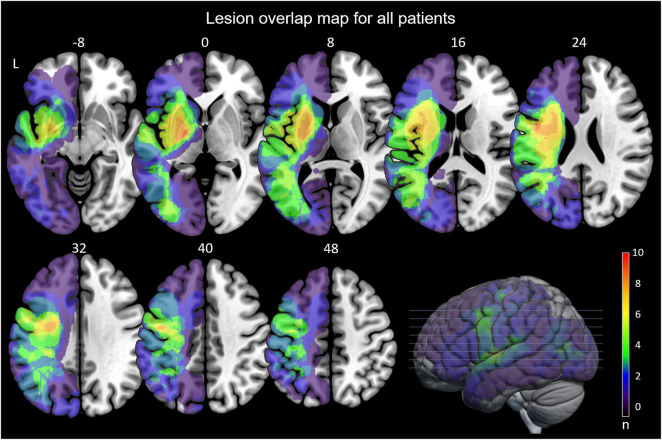
Anatomical overview. Lesion overlap maps for all patients. Numbers are *z*-coordinates given in MNI stereotaxic space from *z* = –8 to z = 48, color coding indicates number of overlapping lesions. L, left, n, number of overlaps.

For an anatomical overview of the lesions see Figure 2. For individual lesion maps see Supplementary Figure 1. Relative lesion volume correlated negatively with BL TULIA performance (*r* = –0.57, 95% CI –0.82 – –0.15, *p* = 0.013) and BL LIMOS (*r* = –0.54, 95% CI –0.82 – –0.15, *p* = 0.022). A pairwise correlation was also found between BL TULIA and BL LIMOS upper limb score (*r* = 0.775, 95% CI 0.09 – 0.79, *p* = 0.021). The correlation between BL TULIA and BL FA values was particularly strong (*r* = 0.53, 95% CI 0.45 – 0.92, *p* < 0.001).

### 3.2 One session of cTBS over IPL-R improved gesturing

A single session of cTBS over IPL-R led to a mean change of 6.24 in ΔTULIA (see Table 2). This effect was more positive than after treatment over IFG-R and sham (mean change of 1.48 and –2.04).

**TABLE 2:** ΔTULIA values.

	Mean ± SD	Range
TULIA baseline score ΔTULIA sham ΔTULIA IPL-R ΔTULIA IFG-R	136.70 ± 65.10 −2.04 ± 8.21 6.24 ± 10.81 1.48 ± 14.16	25–215 −22–10 −8–42 −25–25

IPL-R, right inferior parietal lobe; IFG-R, right inferior frontal gyrus.

**FIGURE 3:**
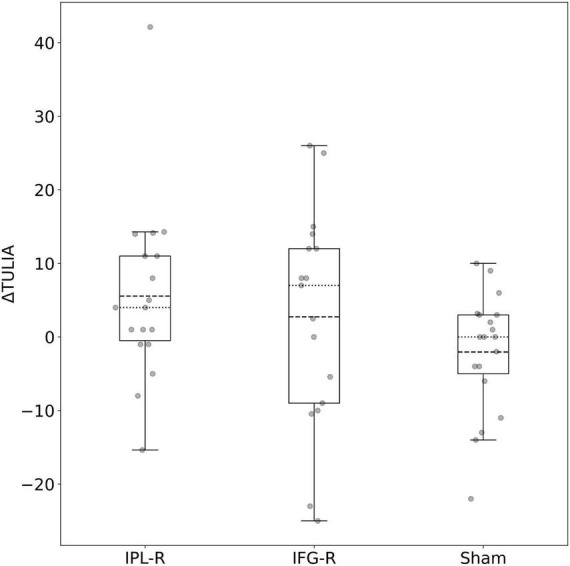
Boxplot of ΔTULIA after IPL-R, IFG-R and sham stimulation. Dashed line: mean. Dotted line: median.

One patient showed an extraordinary improvement after treatment over IPL-R. The mean of ΔTULIA after stimulation over IPL-R was therefore higher than the median. The opposite was observed for IFG-R and sham. All ΔTULIA values with the means and medians are shown in Figure 3.

A linear mixed effects model with fixed effects for treatment, BL FA values and period-specific BL TULIA, adjusted for sequence and period and with a random effect for subject has been fitted in order to explain ΔTULIA as a dependent variable (see Table 3). Pairwise treatment contrasts showed, that, compared to sham, ΔTULIA was higher after stimulation over IPL-R (12.08, 95% CI 6.04 – 18.13, *p* < 0.001). This treatment effect was more pronounced and approximately twice as high as the contrasts for IFG-R vs. sham (6.25, 95% CI –0.20 – 12.70, *p* = 0.058) and IPL-R vs. IFG-R vs. sham (5.83, 95% CI –0.49 – 12.15, *p* = 0.071).

**TABLE 3:** Linear mixed-effects model: The change in praxis skills due to treatment.

			
Variable		(95% CI)	*p*-value

**Treatment (pairwise contrasts)**	**Δ TULIA**		
IPL-R vs. sham	12.08	(6.04–18.13)	*p* < 0.001
IFG-R vs. sham	6.25	(−0.20–12.70)	*p* = 0.058
IPL-R vs. IFG-R	5.83	(−0.49–12.15)	*p* = 0.071

**Covariates**	**Δ TULIA per unit**		

BL TULIA	–0.18	(−0.25–−0.12)	*p* < 0.001
BL FA	140.86	(54.67–227.06)	*p* = 0.001

The estimates are adjusted for sequence and period effects. ΔTULIA per unit refers to the effect of 1 point of BL TULIA or BL FA on ΔTULIA.

BL TULIA are period-specific baseline values whereas BL FA has been measured once per subject at study start.

Sensitivity analyses showed that inclusion of further covariates such as age, sex, baseline scores for JLO, BNT, Novel Tools Test, LIMOS, LIMOS upper limb or relative lesion volume neither improved the model to explain ΔTULIA (in terms of model selection criteria such as Akaike’s and Bayes’ information criterion) nor modified the pattern or magnitude of the estimates in a relevant manner. The robustness of the effect estimation for the pairwise contrast of IPL-R vs. sham was also confirmed in a sensitivity analysis that excluded data from periods in which patients were treated with IFG-R.

### 3.3 FA values in the splenium and TULIA scores at baseline are predictors of gestural outcome after cTBS

The subject-specific baseline FA values and the period-specific TULIA scores before stimulation (see Table 3) were used to determine an adjusted treatment effect. Both estimators revealed further associations though between cTBS and clinical outcome. Firstly, higher baseline FA values of the splenium corporis callosi were predictors of a higher increase in the TULIA score after cTBS over all stimulation sites, and therefore of a better gestural outcome (Table 3, ΔTULIA per BL FA). Secondly, higher TULIA scores at baseline were associated with less overall increase in TULIA, as the negative ΔTULIA per unit shows in Table 3.

## 4 Discussion

In this sham-double-blinded, controlled, proof-of-concept study we investigated the effect of non-invasive brain stimulation on gestural performance in stroke patients. Of the included nineteen left hemispheric stroke patients, almost 85% were apraxic based on a standardized test of gesturing.

As expected, the extent of gestural impairment and everyday upper limb activity was associated with the lesion size (Habegger et al., 2018). Furthermore, the gestural deficit appeared to be outcome relevant as it also correlated with ADL function. Finally, the strong relationship of gestural performance at baseline with measures of the splenial microstructure suggested that intact interhemispheric connectivity contributed in maintaining the gestural production (Viher et al., 2020). The finding is in line with a recent study demonstrating that gestural task performance was better with increased functional connectivity between hemispheres at rest (Watson et al., 2019).

The main goal herein was to explore whether non-invasive brain stimulation may improve gesturing and whether the behavioral effects depend on the integrity of the microstructure in the splenium corporis callosi. We analyzed the effect on gestural performance after cTBS of the non-affected hemisphere, more specifically over the IPL-R, the IFG-R or vertex stimulation (sham). As hypothesized based on the inter-hemispheric rivalry model, we showed that contra-lesional cTBS over IPL-R improved gesture performance, presumably by diminishing the maladaptive inhibition. Furthermore, the gestural responses to cTBS were predicted by the integrity of the splenium as measured by DTI.

There is converging evidence that the IPL-L is a neuronal hub within a broader inferior parietal and premotor limb network and therefore plays an important role in gesturing (Hermsdörfer et al., 2007; Lesourd et al., 2018). Moreover, the left and right IPL is modulable by non-invasive brain stimulation (e.g., rTMS or tDCS) as summarized in a recent scoping review (Pastore-Wapp et al., 2021). So far only two studies investigated the effect of non-invasive brain stimulation on gesturing after left-hemispheric stroke. Bolognini and colleagues demonstrated that anodal tDCS over IPL-L, considered excitatory in nature, improved actual gestural imitation in six left-hemispheric stroke patients (Bolognini et al., 2015). The findings concur with our results, if we assume that gestural improvement by cTBS over IPL-R is mediated by disinhibition of IPL-L. Further in line, a recent study by Ant and colleagues reported that anodal tDCS over left parietal cortex, in addition to standardized motor training, facilitated recovery from imitation deficits in left-hemispheric stroke patients (n = 30) compared to sham stimulation, although overall apraxia score did not improve (Ant et al., 2019). Therefore, along with the present study, NIBS techniques increasingly emerge as an add on treatment option in apraxia, which has a significant impact on the duration and outcome of rehabilitation after stroke (Dovern et al., 2012). The on-going RAdiCS (Rehabilitating stroke-induced Apraxia with direct Current Stimulation) study investigates the effect of anodal (versus sham) tDCS applied over the left posterior parietal cortex (Kleineberg et al., 2020).

In our study, we consider it unlikely that a direct local effect of cTBS may have explained the finding. Based on the role of IPL-R in visual spatial processing (Park, 2017) we would have rather expected the opposite outcome, i.e. the impediment of gesturing, particularly in the imitation domain. Furthermore, the visuospatial deficits controlled by JLO as a covariate did not improve the statistical model.

In favor of a contralateral facilitation of the IPL-L as the main underlying mechanism a recent study of our group demonstrated that cTBS over the IPL-R (either alone or combined with IFG-L) significantly improved gesturing in healthy subjects (Vanbellingen et al., 2020). Furthermore, like the findings herein, the cTBS effects significantly correlated with the FA values of the splenium, particularly in subjects responding well to the treatment. In addition, NIBS studies in either other patient groups or different behavioral domains provide further evidence for an interhemispheric mechanism. Accordingly, cTBS over the IPL-R substantially improved gestural accuracy in patients with schizophrenia (Walther et al., 2019). In an earlier study NIBS improved the motor outcome after stroke through upregulation of the excitability in the lesioned primary motor cortex or through downregulation of the contralateral (healthy) primary motor cortex (Hummel and Cohen, 2006). Similar transcallosal mechanisms were observed in neurocognitive rehabilitation involving NIBS after stroke (Draaisma et al., 2020). Moreover, inhibitory cTBS has been shown to downregulate the hyperexcitability of the contra-lesional hemisphere, thereby restoring the interhemispheric balance and improving neglect (Nyffeler et al., 2008, 2009; Cazzoli et al., 2012) and aphasia (Kindler et al., 2012; Thiel et al., 2013). Intact connectivity of the corpus callosum is critical for the interhemispheric influence of cTBS as shown recently for neglect (Nyffeler et al., 2019). Overall, these studies support the notion that interhemispheric rivalry (Kinsbourne, 1977) can be therapeutically exploited.

We assumed that higher FA values represent areas where fibers are more densely arranged (Zarei et al., 2006) reflecting increased integrity in the microstructure of the corpus callosum that renders the interhemispheric interactions more efficient. In line with this notion, gestural responses to cTBS in the present study and in healthy subjects (Vanbellingen et al., 2020) depend on the microstructural properties of the splenium, connecting the right and left IPL (Hofer and Frahm, 2006). Similarly, behavioral differences in visual exploration after cTBS were predicted by the strength of callosal connectivity (Chechlacz et al., 2015).

The question arises how the observation that the FA values were positively correlated with both the TULIA score at baseline and the change in TULIA score after cTBS over IPL-R can be reconciled with the interhemispheric rivalry model. It is likely that stronger transcallosal connectivity not only favors inhibition of the left IPL, but also the feedback disinhibition of the right IPL. Therefore, at baseline, the released activity of right IPL may have contributed to compensating for gestural deficits in subacute left hemispheric stroke as shown for aphasia (Kindler et al., 2012; Hu et al., 2018). When IPL-R was downregulated by cTBS a transient shift to increased IPL-L activity mediated the gestural improvement more effectively.

The effects of stimulation over the IPL-R were robust as the experimental study design controlled for spontaneous recovery, general rehabilitation and learning effects (Figure 1). The gestural performance was measured twice in 24 hours for each stimulation (IFG-R, IPL-R, sham) and the difference between the two time points was included in the statistical evaluation. Furthermore, the gestural improvement of cTBS over IPL-R was specific as the experimental design included an active control condition (cTBS over IFG-R). In addition, we did not find any behavioral confounding effects either for spatial orientation, visual attention, speech production or for mechanical knowledge. The most compelling explanation for this observation is these tasks involved brain regions outside the stimulation focus of IPL-R and its transcallosal influence on IPL-L. Moreover, there was no significant effect after stimulation of the IFG-R, replicating our findings of a previous study in healthy participants (Vanbellingen et al., 2020). As hypothesized, lesion volume had no influence on gestural improvement after stimulation.

The advantages of cTBS are manifold. It is non-invasive and well tolerated (no pain). The theta burst protocols allowed the application of a high number of magnetic pulses within a single and short application, which is clinically more feasible and for the patients more pleasant than longer protocols. The effects are outlasting the actual stimulation for up to 30 minutes (Nyffeler et al., 2008) providing a time window for behavioral assessment without ongoing stimulation. Finally, if applied in repeated trains (Nyffeler et al., 2019), cTBS may produce long lasting effects (i.e. over months), which is relevant in clinical neurorehabilitation settings.

A limitation of the study is its small sample size. Furthermore, the gestural effects of cTBS were mild. To achieve clinically meaningful and long-lasting effects on gestural function and eventually on ADL, the treatment protocol will likely need multiple cTBS stimulation sessions, as demonstrated for neglect (Cazzoli et al., 2012; Nyffeler et al., 2019) and aphasia (Kindler et al., 2012) or as intended with tDCS for apraxia (Kleineberg et al., 2020). Therefore, randomized clinical trials with multiple cTBS trains are needed to expand on these proof-of-concept results.

## 5 Conclusion

This work is the first double-blinded sham-controlled study of cTBS after stroke with a focus on non-lesioned hemisphere. It describes a promising novel approach to develop add on treatment protocols for neurorehabilitation of stroke patients with apraxia in the subacute phase. The present findings are encouraging as we demonstrated gestural improvements even after a single session of inhibitory cTBS over the IPL-R. The findings were predicted by the microstructural properties of the splenium corporis callosi favoring the transcallosal disinhibition of motor-cognitive functions in the IPL-L as the main mechanism.

## Data availability statement

The raw data supporting the conclusions of this article will be made available by the authors, without undue reservation.

## Ethics statement

The studies involving human participants were reviewed and approved by EKNZ: Ethikkommission Nordwest- und Zentralschweiz. The patients/participants provided their written informed consent to participate in this study.

## Author contributions

MP-W was responsible for patient recruitment, and project coordination provided data collection and analysis, and manuscript writing. DG and DL provided data analysis and manuscript writing. TV gave a critical review of the concept and design of the study. DL, DC, TP, TN, and SW critically revised the manuscript. SP was responsible for patient recruitment and provided data collection. SB provided the concept and design of the study, obtained funding, and critically revised the manuscript conceptualization. All authors assisted in editing and reviewing the submitted manuscript. All authors read and approved the final form for publication.

## Footnotes

1
people.cas.sc.edu/rorden/mricro/index.html

2
http://www.fil.ion.ucl.ac.uk/spm

3
www.nitrc.org/projects/niistat/

4
www.nitrc.org/projects/mricrogl

5
http://dsi-studio.labsolver.org/

## Supplementary material

Supplementary Figure 1 Anatomical overview of individual lesion maps. For each individual patient (1-18), 3D rendering of the lesion in an MNI stereotaxic space is shown. According to the cluster peak, slices on the axial, sagittal and coronal plane were determined to present each individual lesion.
