# Supplementary figures and images for: Improved gesturing in left-hemispheric stroke by right inferior parietal theta burst stimulation
Source: Front Neurosci. 2022 Dec 16;16:998729. doi: 10.3389/fnins.2022.998729 (PMC9800932; doi:10.3389/fnins.2022.998729)

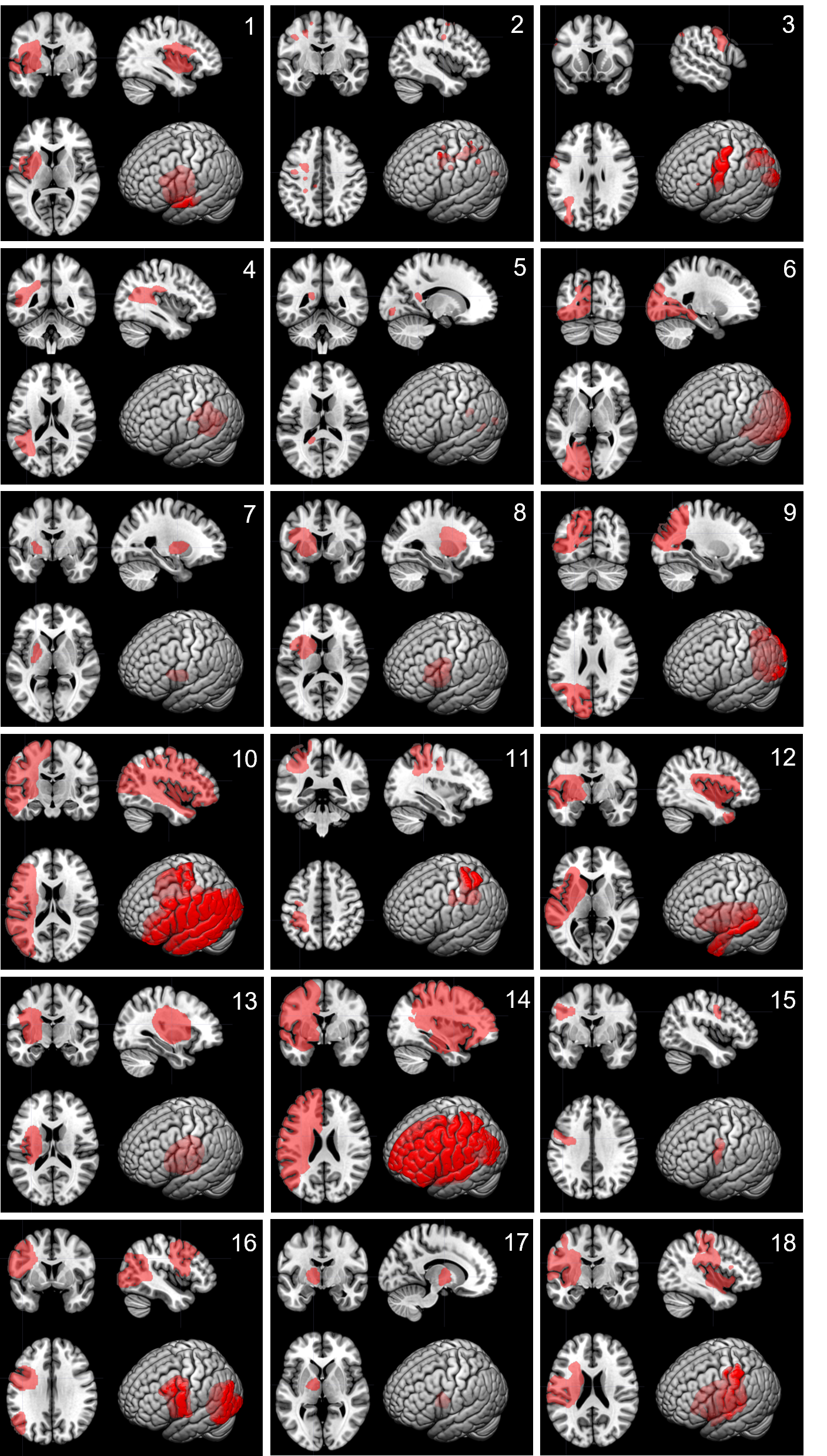

Supplement: Supplementary Figure 1 — Anatomical overview of individual lesion maps. For each individual patient (1-18), 3D rendering of the lesion in an MNI stereotaxic space is shown. According to the cluster peak, slices on the axial, sagittal and coronal plane were determined to present each individual lesion. [file Image_1.TIF]
